# Pneumococci in the African Meningitis Belt: Meningitis Incidence and Carriage Prevalence in Children and Adults

**DOI:** 10.1371/journal.pone.0052464

**Published:** 2012-12-20

**Authors:** Judith E. Mueller, Seydou Yaro, Macaire S. Ouédraogo, Natalia Levina, Berthe-Marie Njanpop-Lafourcade, Haoua Tall, Régina S. Idohou, Oumarou Sanou, Sita S. Kroman, Aly Drabo, Boubacar Nacro, Athanase Millogo, Mark van der Linden, Bradford D. Gessner

**Affiliations:** 1 Agence de Médecine Préventive, Paris, France; 2 Centre Muraz, Bobo-Dioulasso, Burkina Faso; 3 Centre Hospitalier Universitaire Sourou Sanou, Bobo-Dioulasso, Burkina Faso; 4 GNRC Streptococci, Aachen, Germany; Centers for Disease Control & Prevention, United States of America

## Abstract

**Background:**

The development of optimal vaccination strategies for pneumococcal conjugate vaccines requires serotype-specific data on disease incidence and carriage prevalence. This information is lacking for the African meningitis belt.

**Methods:**

We conducted hospital-based surveillance of acute bacterial meningitis in an urban and rural population of Burkina Faso during 2007–09. Cerebrospinal fluid was evaluated by polymerase chain reaction for species and serotype. In 2008, nasopharyngeal swabs were obtained from a representative population sample (1 month to 39 years; N = 519) and additional oropharyngeal swabs from 145 participants. Swabs were evaluated by culture.

**Results:**

Annual pneumococcal meningitis incidence rates were highest among <6-month-old (58/100,000) and 15- to 19-year-old persons (15/100,000). Annual serotype 1 incidence was around 5/100,000 in all age groups. Pneumococcal carriage prevalence in nasopharyngeal swabs was 63% among <5-year-old children and 22% among ≥5-year-old persons, but adding oropharyngeal to nasopharyngeal swabs increased the estimated carriage prevalence by 60%. Serotype 1 showed high propensity for invasive disease, particularly among persons aged ≥5 years.

**Conclusions:**

Serotype 1 causes the majority of cases with a relatively constant age-specific incidence. Pneumococcal carriage is common in all age groups including adults. Vaccination programs in this region may need to include older target age groups for optimal impact on disease burden.

## Introduction

The African meningitis belt is located at the southern border of the Sahara and extends from Senegal to Ethiopia. It is characterized by seasonal hyperendemicity of acute bacterial meningitis during the dry season and sporadic localised and regional epidemics [1], [2]. While meningitis epidemics are exclusively due to meningococci, seasonal hyperendemicity is due to both pneumococci and meningococci [3]–[6]. Pneumococcal meningitis has high incidence among older children and adults with an estimated life time risk of 0.6% and is due primarily to serotype 1 [7].

Several countries in the African meningitis belt, including Burkina Faso, plan to introduce serotype 1 containing pneumococcal conjugate vaccines into their routine infant immunization programs, with the goal to protect children during the first year of life, primarily from pneumonia. Given the high burden of pneumococcal meningitis in older children and adults in the African meningitis belt – and an unknown burden of pneumococcal pneumonia – the question arises whether the vaccination schedule for this region should be adapted to directly protect these age groups from meningitis and other pneumococcal syndromes [7]. This discussion is complex and should include biomedical, financial, logistic and sociological aspects. One approach is to compare vaccination strategies by modeling their impact taking into account age- and serotype-specific incidence, strain transmission (carriage) and vaccine effectiveness. For the African meningitis belt, few serotype-specific estimates of incidence or carriage exist, mainly due to technical difficulties with isolation and serotyping of pneumococci. A new technology of PCR-based serotyping directly on cerebrospinal fluid (CSF) [8] has reduced this barrier.

In this context, we aimed at describing serotype-specific pneumococcal meningitis incidence and carriage prevalence among children and adults in Burkina Faso. We also evaluated whether oropharyngeal added to nasopharyngeal swabbing improved pneumococcal carriage detection.

## Methods

### Ethics Statement

We obtained ethical approval for the carriage and surveillance studies from the Burkina Faso national review committee and the Centre Muraz ethics committee.

### Surveillance

During March 2007 to December 2009, we conducted a hospital-based bacterial meningitis surveillance study in four administrative health districts in and around Bobo-Dioulasso, Burkina Faso. During March 2007-February 2008, we surveyed the 588,022 inhabitants of the urban zone; during March 2008-December 2009, and following additional funding, we expanded the surveyed population to the 900,176 inhabitants of both the urban and rural zones. The latter represented about 6% of the country’s population. As previously reported [9], all suspected meningitis patients of the surveyed population presenting to any health care service in the surveyed zone were supposed to receive a lumbar puncture. The study zone included the country’s second largest university hospital, which is the major reference center for the Western region of Burkina Faso, all district hospitals and all health centers; health centers performed lumbar puncture and provided clinical care, but did not provide in-patient care for meningitis patients. An urban private clinic did not participate in the surveillance, but reportedly saw few suspected meningitis cases. All CSF samples were analyzed using a multiplex PCR technique for identification and serotyping of pneumococcal meningitis cases at Centre Muraz in Bobo-Dioulasso. This involved DNA extraction by a cycle of freezing and thawing and centrifugation, gene amplification of *lytA* for *Streptococcus pneumoniae* (Sp) [10], [11], and, if a pneumococcal etiology was identified, an algorithm of 29 primers screening for 43 serotypes or -groups (see Supporting Information, Table S1) [8]. In addition, culture and latex agglutination [12] were performed on CSF samples that arrived in the laboratory within three hours after lumbar puncture. Pneumococci isolated in this case underwent serotyping by PCR [8], as well. For quality control, all pneumococcal isolates were confirmed and serotyped at the German Reference Center for Streptococci (Aachen) by Neufeld Quellung reaction using antisera provided by the Statens Serum Institute (Copenhagen, Denmark) [13]; similarly, a subset of CSF samples was tested for pneumococcal etiology and serotype by PCR [8].

### Carriage Study 2008

During February 2008, we recruited by cluster sampling a representative sample (N = 519) of the urban population in Bobo-Dioulasso aged 1 month to 39 years. Ten out of the town's 22 neighborhoods were selected randomly, and between 22 and 24 crossroads in each neighborhood were randomly chosen as starting points. At each starting point, the street of recruitment was randomly determined and the compounds along this street were visited, starting with the first compound after the crossroad. In each compound, a list of inhabitants aged 1–11 months, 1–9 years, 10–19 years, 20–29 years and 30–39 years was established and one participant randomly chosen for each age group. If the chosen person refused participation or if the age group was not represented in the compound, the closest neighboring compound was visited. Persons with residence outside urban Bobo-Dioulasso, non-African ethnicity, or severe disease, including hemophilia, were excluded.

Following written, informed consent, a baseline questionnaire was administered that determined medical history and life style of participants and their families. During February 21 and March 5, 2008, all participants visited the Centre Muraz outpatient clinic, where the questionnaire was completed, and weight and height were measured for children less than ten years old using available clinic scales. For children shorter than 121 cm, malnutrition was defined as weight-for-height below the third percentile according to WHO child growth standards [14]. Swabbing was then performed by ear-nose-and-throat specialist nurses. Nasopharyngeal swabs via the nose were taken from all 519 participants using flexible sterile swabs with a calcium alginate tip. In addition, for a representative sub-sample of 145 participants aged 1 to 39 years, we also took oropharyngeal swabs from the posterior pharyngeal wall via the mouth using cotton-tipped sterile swabs [15]. Isolation and identification of pneumococci followed the WHO consensus recommendations where possible [16]. Swabs were immediately streaked onto sheep blood agar with 7% gentamycine. The dishes were stored immediately in a 5%-CO_2_ atmosphere at room temperature for a maximum of two hours until incubation at 37°C. From each incubated plate with colonies suspicious of *S. pneumoniae* by aspect (alpha-hemolysis, umbilicated), three bacterial colonies were selected from distinct locations on the plate. They were submitted to optochine, Gram stain and catalase. Colonies with an optochine diameter ≥14 mm, or an optochine diameter of 8–13 mm and positive bile solubility, were purified and prepared for transport on Portagerm AMIES® agar swabs (Biomérieux) or storage on Brain-Heart-Infusion and glycerol at −80°C. All isolated pneumococci were confirmed and serotyped at the German Reference Center for Streptococci using standard techniques and Neufeld Quellung [13].

### Statistical Analyses

We used standard statistical methods, including adjusting for the design effect of cluster sampling. Incidence rates and 95%-confidence intervals were calculated using national census data (2006) extrapolated to the surveillance years and assuming a Poisson distribution. Differences in participant characteristics by nasopharyngeal carriage status were tested by logistic regression while adjusting for age. To assess the theoretical propensity of individual serogroups to cause invasive disease given carriage, relative to other serogroups, we calculated serotype-specific invasiveness indexes as odds ratios (II_OR_ = ad/bc) where a = n cases(serotype), b = n cases(other serotypes), c = n carriers(serotype), d = n carriers(other serotypes) [17]. Exact 95% confidence intervals were calculated, using Cornfield approximation in case of empty cells. All analyses were done in STATA version 11.

## Results

### Meningitis Incidence

During March 2007-December 2009, we enrolled 1,008 suspected meningitis cases, of which CSF was analyzed by PCR for 976 (97%), by culture for 983 (98%) and by latex for 927 (92%), and 999 (99%) had at least one of these tests. Among the 454 confirmed bacterial cases (45%), we identified 159 (35%) pneumococci (32 confirmed by PCR only, 8 by culture only and 8 by latex only), 286 (63%) meningococci and 9 (2%) *Haemophilus influenzae.* One hundred twenty-seven pneumococcal cases (80%) had pneumococcal serotyping performed. Among the 82 culture-positive pneumococcal cases, 25 pneumococci were stored and later serotyped by PCR or Quellung. No discordance in serotype result was found for cases tested by more than one method.

We calculated annual incidence rates per 100,000 inhabitants over two entire years from March 2007 to February 2009 to avoid any bias from seasonal variation. Overall annual pneumococcal meningitis incidence was 8.9 (95%-CI, 7.5–10.6), 7.3 (6.0–8.8) for serotyped cases, 3.7 (2.8–4.8) for serotype 1, and 4.7 (3.7–6.0) and 4.9 (3.8–6.1) for serotypes included in PCV-10 and PCV-13, respectively (**Table 1**). Infants aged <6 months had the highest annual incidence rate of pneumococcal meningitis (58/100,000, **Table 1**) but a second peak was observed among 15- to 19-year-old persons (15/100,000). These two peaks also were found for PCV-10 or PCV-13 serotypes. Serotype 1 meningitis incidence varied little by age with the highest rate found among 15- to 19-year-old persons (8/100,000). No systematic difference in incidence existed for urban versus rural populations. Sixty-seven percent of any pneumococcal cases (77% of cases preventable by PCV-10 or -13) occurred among ≥5-year-old persons.

**Table 1:** Age-specific annual incidence rates of pneumococcal meningitis, by group of serotype, Bobo-Dioulasso, Burkina Faso, March 2007–February 2009.

	0–5 mo(N = 34,782)	6–11 mo(N = 27,826)	1–4 yrs(N = 201,240)	5–9 yrs(N = 225,836)	10–14 yrs(N = 225,836)	15–19 yrs(N = 89,211)	20–29 yrs(N = 178,422)	30–39 yrs(N = 129,630)	≥40 yrs(N = 388,890)	Total(N = 1,501,672)
Any pneumococcal	57.5 (35.1–88.8) [20]	43.1 (22.3–75.3) [12]	5.0 (2.4–9.1) [10]	10.6 (6.8–15.8) [24]	8.9 (5.4–13.7) [20]	14.6 (7.8–24.9) [13]	5.6 (2.7–10.3) [10]	6.2 (2.7–12.2) [8]	4.4 (2.6–7.0) [17]	8.9 (7.5–10.6) [134]
Any serotype (if serotyped)	54.6 (32.9–85.3) [19]	39.5 (19.7–70.7) [11]	3.5 (1.4–7.2) [7]	7.1 (4.1–11.5) [16]	7.1 (4.1–11.5) [16]	14.6 (7.8–24.9) [7]	3.9 (1.6–8.1) [7]	3.1 (0.8–7.9) [4]	4.1 (2.4–6.7) [16]	7.3 (6.0–8.8) [109]
Serotype 1	5.8 (0.7–20.8) [2]	3.6 (0.09–20.0) [1]	1.0 (0.1–3.6) [2]	6.2 (3.4–10.4) [14]	5.8 (3.1–9.8) [13]	7.9 (3.2–16.2) [7]	3.4 (1.2–7.3) [6]	2.3 (0.5–6.8) [3]	2.1 (0.9–4.1) [8]	3.7 (2.8–4.8) [56]
10-valent vaccine types *	14.4 (4.7–33.6) [5]	25.2 (10.0–51.8) [7]	2.0 (0.5–5.1) [4]	6.2 (3.4–10.4) [14]	5.8 (3.1–9.8) [13]	9.0 (3.9–17.7) [8]	3.9 (1.6–8.1) [7]	3.1 (0.8–7.9) [4]	2.3 (1.1–4.4) [9]	4.7 (3.7–6.0) [71]
13-valent vaccine types†	14.4 (4.7–33.6) [5]	25.2 (10.0–51.8) [7]	2.5 (0.8–5.8) [5]	6.2 (3.4–10.4) [14]	5.8 (3.1–9.8) [13]	9.0 (3.9–17.7) [8]	3.9 (1.6–8.1) [7]	3.1 (0.8–7.9) [4]	2.6 (1.2–4.7) [10]	4.9 (3.8–6.1) [73]
Theoretical serotype coverage with 10- (13-) valent vaccine (%)	26 (26)	64 (64)	57 (71)	87 (87)	82 (82)	62 (62)	100 (100)	100 (100)	56 (63)	65 (67)

Serotyping based on PCR on CSF [8] or Quellung reaction on isolates (if strain isolated and stored).

Rates are expressed per 100,000 inhabitants (95% confidence interval) [number of cases].

* includes serotypes 1, 4, 5, 6B, 7F, 9V, 14, 18C, 19F and 23F.

† includes serotypes 1, 3, 4, 5, 6A, 6B, 7F, 9V, 14, 18C, 19A, 19F and 23F.

**Figure 1:**
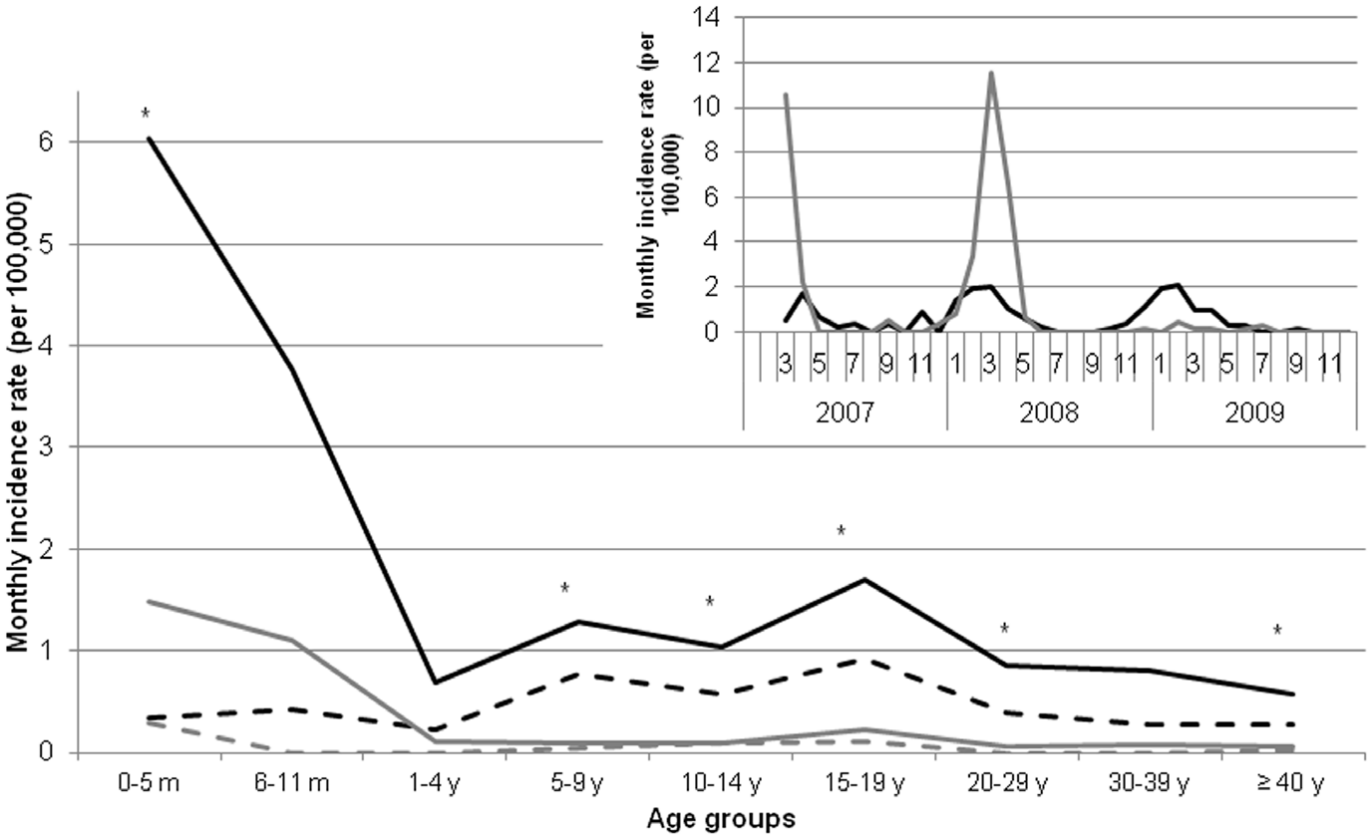
Monthly incidence rates of bacterial meningitis in Bobo-Dioulasso, Burkina Faso, March 2007 to December 2009. a) Pneumococcal (black line) and meningococcal (grey line) meningitis. b) Age-specific rates of pneumococcal meningitis, by season. Black lines, dry season. Grey lines, rainy season. Full lines, all pneumococcal cases. Dashed lines, serotype 1 cases only. Asterixes, 95% confidence intervals of incidence rates of all pneumococcal cases do not overlap between dry vs. rainy season, suggesting statistical significance of difference.

During the dry season months of December-May, the monthly incidence rates per 100,000 population of any pneumococcal and serotype 1 meningitis were 1.1 (95% CI, 0.9–1.3) and 0.4 (0.3–0.6) while during the wet season months of June-November, incidence rates were 0.1 (0.1–0.2) and 0.0 (0.0–0.1) (**Figure 1**). The ratio of the average monthly incidence during dry compared to rainy seasons was 9.1 (95%-CI 3.9–26.0) for serotype 1 meningitis and 7.5 (3.6–18.2) for other serotypes, without variation by age group. Serotype 1 showed a flat age distribution during both the dry and the rainy season (**Figure 1** **,** **Table 1**).

The case fatality ratio from presentation to the end of health center care was 40% (62/155), without substantial variation by season, age or serotype, including serotype 1 (27/61 cases). Among survivors at hospital discharge, a motor or sensory handicap was identified for 9 of 28 (32%) <5-year-old children and for 3 of 65 (5%) ≥5-year-old persons (Chi-square test for difference in proportion, *P*<0.001).

**Figure 2:**
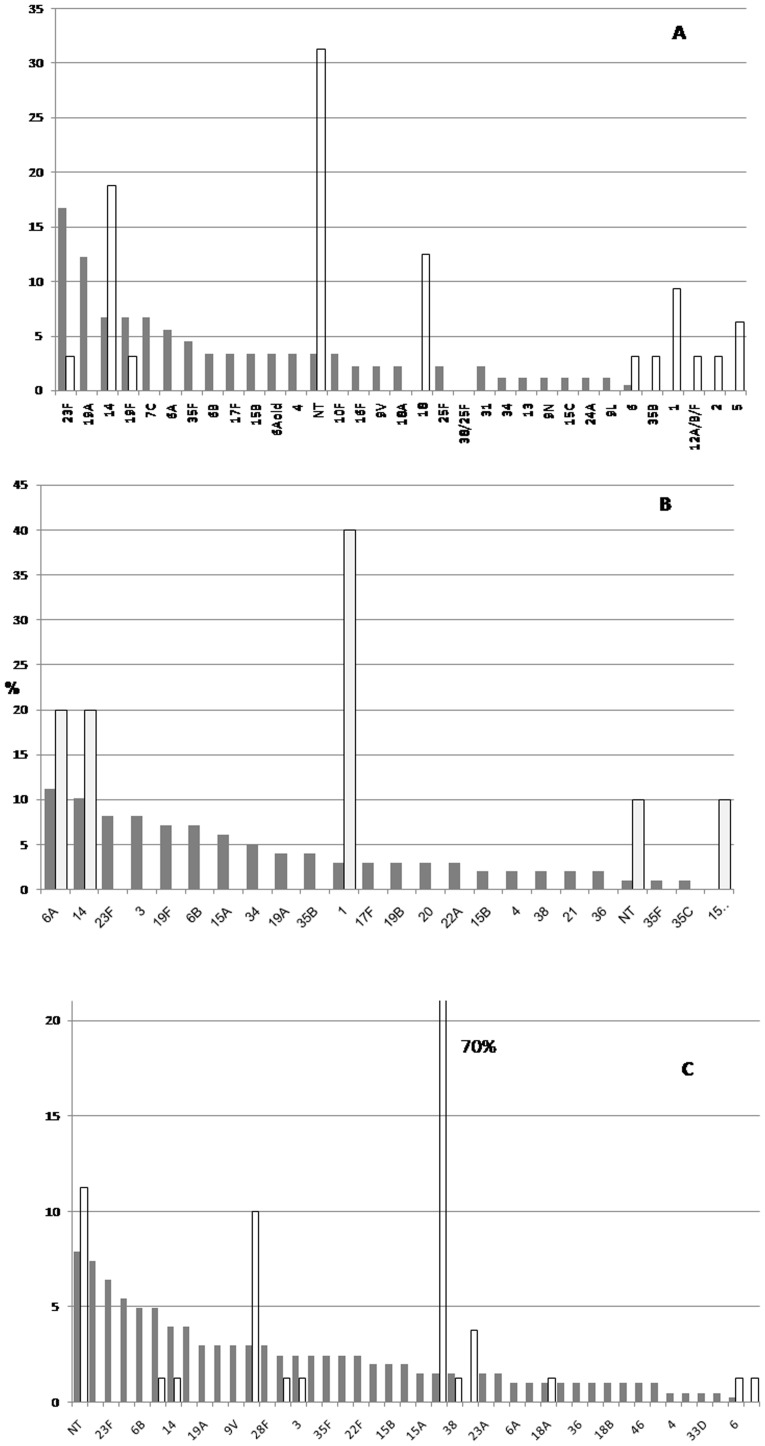
Serotype or -group distribution among carriage (dark gray) and pneumococcal cases (light gray), Burkina Faso, 2007–9. Serotyping based on cerebrospinal fluid polymerase chain reaction testing [8] or Quellung reaction on invasive isolates; and on Quellung reaction for carriage isolates. Each panel A–C shows only serotypes that were found in this age category. A: children <1 years of age, 92 nasopharyngeal carriage and 32 cases. B: children 1–4 years of age, 98 carriage isolates (90 nasopharyngeal, 8 oropharyngeal) and 10 cases. C: children ≥5 years of age and adults, 203 carriage isolates (157 nasopharyngeal, 46 oropharyngeal) and 84 cases. NT, invasive isolates with serotypes not included in the PCR serotyping algorithm (29 primers); or carriage isolates yielding negative results in the Quellung reaction panel.

The most commonly identified serotypes or serogroups among children <5-years-old were 14 (19% of serotyped cases), 1 (16%) and 18 (8%), with 22% being non-typable (i.e., serotypes not included in the PCR algorithm); among persons ≥5-years old, the most common serotypes were 1 (70%) and 12F (10%), with 11% being non-typable (**Figure** 2**a–c**).

### Carriage

Among the 519 participants of the carriage study, clinical rhinitis at swabbing was common (29% of children age <5 years, 11% of persons age ≥5 years), 22% of children <121 cm tall had malnutrition, 8% of ≥15-year-old persons smoked tobacco, 40% of ≥10-year-old persons were illiterate and two-thirds of participants had a television set in the compound (see Supporting Information, Table S2). Any pneumococcal carriage was found in 166 (32%) participants and nasopharyngeal carriage prevalence decreased from 73% among 6- to 11-month-old children to 11% among 25- to 29-year-old adults (**Table 2**). Nasopharyngeal carriers of pneumococcus were younger than non-carriers (mean, 9.1 versus 18.2 years, Student’s t-test, *P*<0.001), while other characteristics of life style or medical history did not differ significantly between carriers and non-carriers after adjustment for age. The carriage prevalences for any pneumococci, 10-valent vaccine serotypes, and 13-valent vaccine serotypes, were 63%, 23% and 35%, respectively, among <5-year-old children and 22%, 6% and 7%, respectively, among ≥5-year-old persons (**Table 2**).

**Table 2:** Age-specific prevalence of pneumococcal carriage, by serotype group, Bobo-Dioulasso, Burkina Faso, February 2008.

Age groups (N)										
	0–5 mo(N = 32)	6–11 mo (N = 30)	1–4 yrs (N = 66)	5–9 yrs (N = 65)	10–14 yrs (N = 65)	15–19 yrs (N = 66)	20–24 yrs(N = 64)	25–29 yrs(N = 65)	30–39 yrs (N = 66)	Total (N = 519)
Documented nasopharyngeal carriage prevalence										
Any pneumococci (%)	66 (48–83)	73 (57–90)	58 (45–70)	35 (23–47)	31 (19–42)	21 (11–31)	14 (5–23)	11 (3–19)	18 (8–28)	32 (28–36)
10-valent vaccine types * (%)	22 (7–37)	27 (10–43)	21 (11–31)	11 (3–19)	8 (1–14)	5 (0–10)	3 (0–8)	3 (0–7)	6 (0–12)	10 (8–13)
13-valent vaccine types† (%)	31 (14–48)	47 (28–66)	32 (20–43)	14 (5–22)	9 (2–16)	8 (1–14)	3 (0–8)	3 (0–7)	8 (1–14)	14 (11–18)
Non 13-valent vaccine types† (%)	34 (17–52)	33 (15–51)	29 (18–40)	23 (13–34)	22 (11–32)	15 (6–24)	11 (3–19)	9 (2–16)	11 (3–18)	19 (16–23)
Estimated overall nasopharyngeal plus oropharyngeal carriage prevalence‡										
Extrapolation factor	1.3§	1.3§	1.3	1.4	1.4	2.0	2.0	2.1	2.1	1.6
Any pneumococci (%)	84 (67–95)	97 (83–100)	77 (65–87)	51 (38–63)	45 (32–57)	42 (30–55)	28 (18–41)	23 (14–35)	39 (28–52)	49 (45–53)

Nasopharyngeal swabs were taken from all 519 participants and additional oropharyngeal swabs were taken from a subset of 145 participants aged 1 to 39 years. Serotyping based on Quellung reaction.

Prevalence percent (95% confidence interval).

* includes serotypes 1, 4, 5, 6B, 7F, 9V, 14, 18C, 19F and 23F.

† includes serotypes 1, 3, 4, 5, 6A, 6B, 7F, 9V, 14, 18C, 19A, 19F and 23F.

‡ An extrapolation factor was calculated for each age group as the ratio of pneumococcal carriage at any site to pneumococcal carriage in the nasopharynx.

§ Children <12 months had only naso-pharyngeal swabs, thus no specific extrapolation factor could be calculated. We assumed the factor to be similar as in 1- to 4-year-old children.

**Figure 3:**
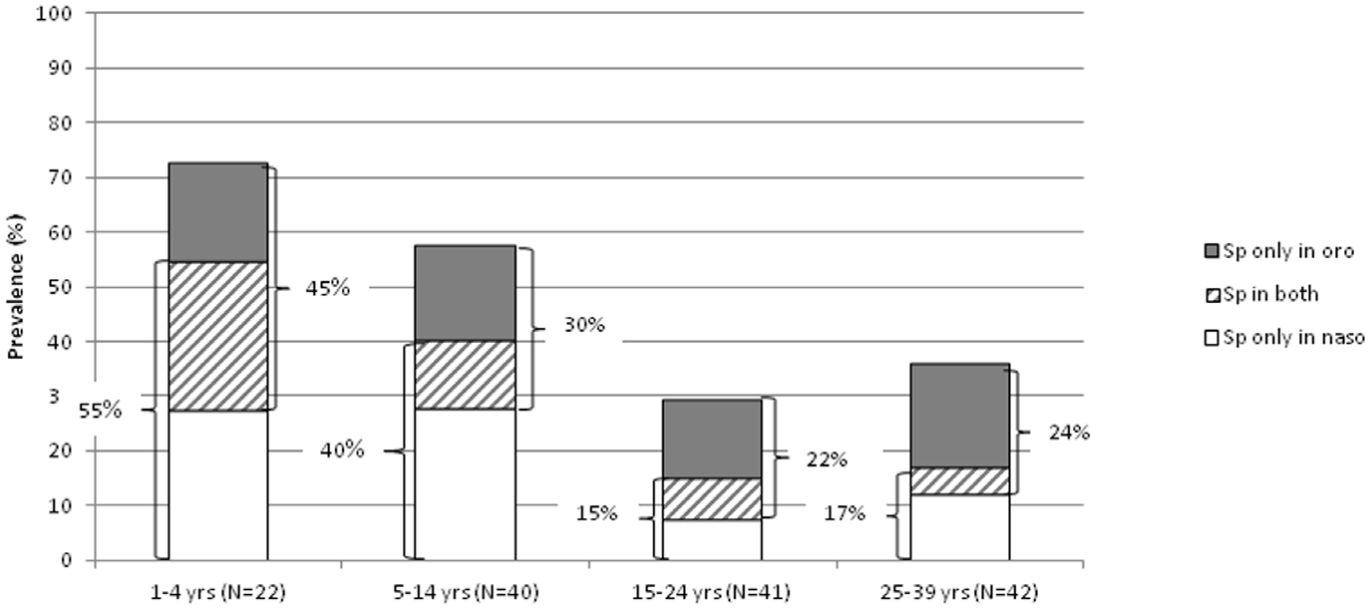
Age-specific pneumococcal carriage prevalence by carriage site in pharynx among 145 participants with both naso- and oropharyngeal swabs. Bobo-Dioulasso, 2008.

Among the 145 1- to 39-year-old participants with additional oropharyngeal swabbing, 25 (17%) were exclusively nasopharyngeal carriers and 25 (17%) exclusively oropharyngeal carriers, while 16 (11%) carried at both sites (**Figure 3**). Among 1- to 14-year-old children, nasopharyngeal compared to oropharyngeal prevalence was 10% higher, while among 15- to 39-year-old persons, oropharyngeal compared to nasopharyngeal prevalence was 7% higher. Adding a second technique increased documented carriage prevalence 1.6-fold from 28% (95% CI, 21%–36%) to 46% (37%–54%). To use these data to estimate overall carriage prevalence in our study population, for each age group we multiplied the nasopharyngeal carriage prevalence by the extrapolation factor, with the latter defined as “(nasopharyngeal+oropharyngeal)/nasopharyngeal carriers”. For <1-year-old children, we assumed this factor to be the same as among 1- to 4-year-old children. Using this method, the extrapolated overall carriage prevalence ranged from 97% among 6- to 11-month-old children to 23% among 25- to 29-year-old adults (**Table 2**).

From the 191 persons with oropharyngeal or nasopharyngeal carriage identified, 406 pneumococcal isolates were obtained. Among these 406 isolates, 391 from 180 carriers were serotyped, including 336 from nasopharyngeal and 55 from oropharyngeal swabs. Among nasopharyngeal carriers of pneumococci, 19% had one, 43% two and 37% three colonies positive for pneumococcus; these figures were 47%, 24% and 29% among oropharyngeal carriers. Of the 180 carriers, 43 were age <1 year, 41<1–4 years, and 96≥5 years; within these three age groups, carriage of two serotypes was observed for 3 (7%), 3 (7%) and 11 (11%) persons, respectively and no participants had more than two serotypes identified. Among patients who had an additional oropharyngeal swab, two serotypes were found in 17% of participants versus 8% of participants who did not have an oropharyngeal swab (difference *P* = 0.039). Among persons with a nasopharyngeal swab, two serotypes were found in 0%, 9% and 5%, respectively, of participants with 1, 2 and 3 nasopharyngeal isolates retrieved (difference *P* = 0.335); among persons who also had an oropharyngeal swab, two serotypes were found in 43%, 75% and 20%, respectively, of participants with 1, 2 and 3 oropharyngeal isolates (difference *P* = 0.255).

Among <1-year-old participants, the most frequently observed serotypes (carried by at least 5% of participants) were 23F, 19A, 7C, 6A and 19F, which together accounted for 51% of carriers. Among 1- to 4-year-old participants, these figures were 19F, 23F, 6A and 6B, accounting for 42% of carriers, and among 5- to 39-year-old carriers, these were non-typable strains, 16F, 6B, 23F, and 7C, accounting for 34% of carriers. These distributions were similar if unit of observation was bacterial isolates rather than persons with carriage (**Figure 2a–c** **)**. Serotype 1 carriage was found in one 4- and one 10-year-old child. Among persons 5–39 years old (numbers insufficient for younger children), the following serotypes were significantly (*P*<0.05) more frequent among oropharyngeal (N = 46) than nasopharyngeal (N = 157) isolates: 23A (7% vs. 0%), 6B (13% vs. 3%) and NT (16% vs. 6%).

A significantly higher theoretical relative propensity for invasive disease (ie, the lower 95% confidence limit of the invasiveness index was >1.00) was found for serotypes 1 (II_OR_ 83.5, 95% confidence interval 20.6–716.6), 5 (∞; 1.2-∞), 12A/B/F (4.7; 1.1–24.5) and non-typable pneumococci (2.6; 1.1–6.1) (**Table 3**). The II_OR_ of serotype 1 was 12.8 (1.4–596.1) for <5-year-old children and 146.9 (22.0–5991.3) for ≥5-year-old persons.

**Table 3:** Propensity of pneumococcal serotypes for invasive disease (meningitis) given carriage, relative to other serotypes or -groups.

Serotypeor -group	N Cases due to this Serotype	N Cases due toOther Serotypes	N Carriers ofthis Serotype	N Carriers ofOther Serotypes	Invasiveness Index as Odds Ratio (95% Confidence Interval)
1	57	56	2	164	83.5 (20.6, 716.6)
2	1	112	0	166	∞‡
3	1	112	4	162	0.3 (0.01, 3.7)
4	0	113	2	164	0 (0, 2.8)
5	3	110	0	166	∞ (1.2, ∞)
6 *	4	109	20	146	0.3 (0.1, 0.8)
7C	0	113	10	156	0 (0, 0.5)
9V	0	113	3	163	0 (0, 1.9)
12A/B/F†	9	104	3	163	4.7 (1.1, 27.5)
13	0	113	3	163	0 (0, 1.9)
14	6	107	10	156	0.9 (0.3, 2.8)
15A	0	113	4	162	0 (0, 1. 4)
15B/C *	1	112	6	160	0.2 (0.01, 2.0)
16F	0	113	6	160	0 (0, 0.9)
17F	0	113	6	160	0 (0, 0.9)
18 *	4	109	8	158	0.7 (0.2, 2.8)
19A	0	113	9	157	0 (0, 0.6)
19B	0	113	5	161	0 (0, 1.1)
19F	1	112	11	155	0.1 (0.0, 0.9)
22F	0	113	3	163	0 (0, 1.9)
23F	1	112	18	148	0.07 (0.0, 0.5)
34	0	113	5	161	0 (0, 1.1)
35B	0	113	2	164	0 (0, 2.8)
35F	0	113	5	161	0 (0, 1.1)
38/25F *	3	110	3	163	1.5 (0.2, 11.3)
48	1	112	0	166	∞‡
NT	19	94	12	154	2.6 (1.1, 6.1)

The invasiveness index was calculated using all cases of pneumococcal meningitis with serotype or -group information identified during the dry season months of 2007–2009 (N = 113) and pneumococcal carriage (N = 166) identified in Bobo-Dioulasso, Burkina Faso, March 2007– February 2009. Serotyping was based on cerebrospinal fluid polymerase chain reaction testing [8] or Quellung reaction on invasive isolates; and on Quellung reaction for carriage isolates. The table reports serotypes that were found in at least one meningitis case, found in at least three carriers, or included in 10- or 13-valent pneumococcal conjugate vaccines.

* Discrimination within the serogroup or between serotypes not possible for some meningitis cases due to PCR algorithm.

† PCR serotyping on invasive isolates or CSF cannot discriminate between 12A/B/F, while Quellung typing of carriage isolates identified 12F only.

‡ Cornfield estimation of the confidence interval possible only under certain conditions.

## Discussion

This study in the African meningitis belt found that over 77% of vaccine-preventable cases occurred among ≥5-year-old persons and that the incidence rate of serotype 1 meningitis was constantly high across all age groups. Applying standard microbiological techniques (bile solubility testing and Quellung serotyping) and conventional nasopharyngeal swabbing, we found relatively high carriage prevalence among ≥5-year-old persons and a 60% increase in carriage prevalence estimates when an oropharyngeal swab was added.

Our incidence estimates for overall pneumococcal meningitis are comparable to other periods or places in the meningitis belt [3], [6], [7], [18], although they are in the lower range for <1-year-old infants and for the elderly. Both for serotype 1 and other serotypes, hyperendemic incidences during the dry season were almost ten-fold higher compared to endemic incidences during the rainy season, while this endemic incidence was comparable in magnitude and age distribution to the annual incidence rate of pneumococcal meningitis in Europe [19]. Few studies have investigated the impact of climate in the meningitis belt on pneumococcal disease. One explanation could be seasonal variation of pneumococcal transmission. Only one study from The Gambia reported a two-fold increase in carriage prevalence across seasons, however without specifying the months and associated climate [20]. For meningococci, some evidence suggests that the hyperendemic increase of incidence during the dry season [10- to 100-fold (**Figure 1**)] is due to increased risk of invasive disease given colonization rather than increased bacterial transmission [2]. The damage conferred to the pharyngeal mucosal barrier by persisting extremely low air humidity or high air dust load is a potential explanation, and this model may also apply to pneumococci. In theory, this could have implications for vaccine protection. If a damaged mucosal barrier allows direct invasion from the pharynx into the meninges, as previously hypothesized [2], [21], then bacteria could bypass serum immune defense, and the principal action of vaccine-induced protection would occur on the mucosal surface. Such a hypothesis underlines the importance of evaluating thoroughly the effective impact of meningococcal and pneumococcal conjugate vaccine introduction in the African meningitis belt.

Similar to serogroup A meningococci, serotype 1 carriage is rarely detected except during outbreaks [22]. Given the high incidence rates we observed in our study, serotype 1 must have circulated relatively constantly in our study population, while rarely forming colonies in the pharynx or alternatively forming colonies that are difficult to detect by standard swabbing methods. In a study among 1- to 39-year-old Burkinabè, we previously found that seroprevalence of IgG antibody concentrations ≥0.35 µg/ml against serotype 1 increased with age from 20% to 70%, similar to other serotypes [23]. In this context, the finding that serotype 1 incidence does not decrease after infancy despite increasing antibody concentrations may indicate that adults fail to acquire functional antibody. This is consistent with a theoretical relative propensity for invasive disease of serotype 1 that was comparable to reports for children under age five years on other continents [15], [24]. One should keep in mind that relative propensity as a proxy for invasiveness provides limited evidence due to its theoretical and ecological nature, as it is not based on individual patient data. In any case, the epidemiology of pneumococcal meningitis in the meningitis belt likely results from a complex interaction between strain biology and competition, climate impact on host and bacteria, population mixing patterns and hygienic conditions, and other still unknown factors.

We likely underestimated incidence, as a substantial number of patients may not have presented for medical care, particularly infants with a non-specific clinical presentation [4]. Although PCR-based serotyping of CSF increased the proportion of serotyped cases compared to Neufeld Quellung, the PCR algorithm was limited, at the time of our study, to 43 pre-selected serotypes or -groups, classifying all other cases as non-typable. In control analyses with Quellung, serotypes 2, 13, 46 and 48 were identified among such non-typable cases [8] and they are historically rare serotypes. However, as many as 30% of pneumococcal meningitis cases among children <1 year were classified as non-typable, and non-typable pneumococci showed a high theoretical relative propensity for invasive disease, emphasizing the need to periodically identify the involved serotypes by Quellung and to regularly update the PCR algorithm (see http://www.cdc.gov/ncidod/biotech/strep/pcr.htm for primer updates).

To our knowledge, this is the first study reporting pneumococcal carriage estimates specifically for the meningitis belt and we found high pneumococcal carriage prevalence. In the Gambia, nasopharyngeal swabbing studies [20] described point prevalence of around 80% among <1-year-old children and up to 25% among their mothers, and Hill [25] reported prevalence ranging from 95% in infants to 50% in adults ≥40 years of age. Similar to this latter study, we could not identify any risk factor for pneumococcal carriage other than age.

We found that nasopharyngeal swabbing alone underestimated pneumococcal carriage prevalence in all age groups by at least 30% and that oropharyngeal compared to nasopharyngeal swabbing yielded higher prevalence in teenagers and adults, which is in concordance with a study on Native American adults [26]. Nasopharyngeal swabbing alone appeared to underestimate particularly the prevalence of serotypes 23A, 6B and non-typable pneumococci. In our study, adding oropharyngeal to nasopharyngeal swabbing had a greater effect on detection of multiple serotype carriage than the number of colonies evaluated (the overall frequency of multiple carriage was comparable to studies in Europe [27]). This may be different if >10 colonies were evaluated and if inexperienced technicians read the plates, but the fact that picking several colonies with similar morphology does not increase serotype yield has been described for PCR serotyping of carriage colonies [28]. Carriage studies in this region should use both naso- and oropharyngeal swabs, whenever logistically and ethically feasible. Recently developed techniques of broth enrichment culture and PCR-based pneumococcal detection may further increase the estimated carriage prevalence [28].

A major component of the public health benefit of PCVs is the indirect protection of unvaccinated persons due to reduced transmission following vaccine introduction [29]. In this setting where ≥5-year-old persons experience 77% of PCV-preventable meningitis burden, mostly due to serotype 1, and frequently carry pneumococci, a vaccination strategy targeting only <5-year-old children may not provide substantial indirect protection, if younger children respond sub-optimally to serotype 1 vaccine or if serotype 1 circulation among older children and adults does not depend on carriage among younger children. If this hypothesis is true, the vaccination strategy could be adapted by adding catch-up campaigns in older age groups. It also will be important to continue to monitor serotype distribution, as even among younger children many identified serotypes are not covered in current vaccine formulations. The presented data are useful for populating mathematical models predicting the impact of such different PCV strategies on disease burden. Pneumococcal pneumonia incidence estimated specifically for the meningitis belt would be of great value, as high meningitis incidence and carriage prevalence in adults may or may not translate into high pneumonia incidence [7]. Preliminary data from Northern Togo suggest that pneumococcal pneumonia is similarly seasonal and of high incidence in older age groups [30]. Overall, the presented data underline that the understanding of pneumococcal pathophysiology in the African meningitis belt should be improved to optimize vaccine impact.

## Supporting Information

Table S1 **Algorithm of sequential multiplex polymerase chain reaction testing (PCR) for** ***Streptococcus pneumoniae*** **serotype determination for meningitis belt countries, with and internal positive control using** ***cps*** **A in each reaction.**
(DOCX)

Table S2 **Characteristics of carriage study participants aged 1 month to 39 years (N = 519), by nasopharyngeal pneumococcal carriage status. Bobo-Dioulasso, February 2008.**
(DOCX)
