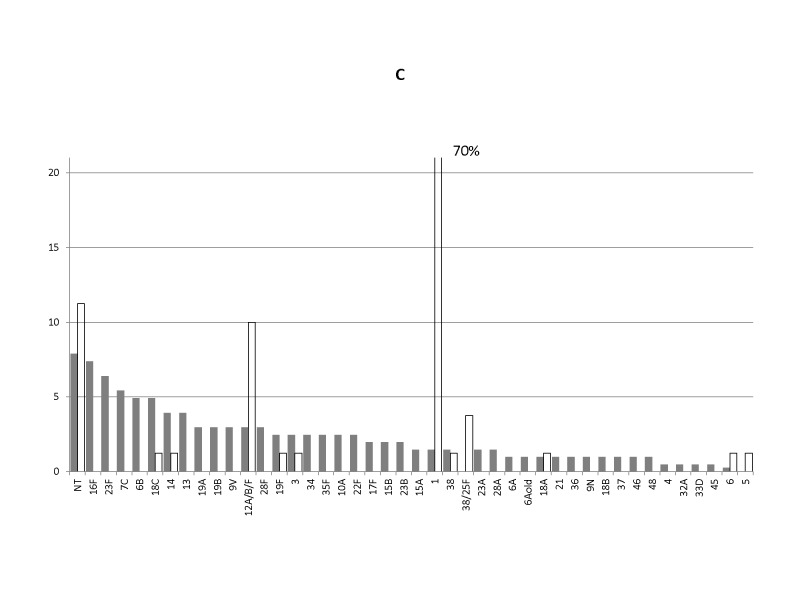# Correction: Pneumococci in the African Meningitis Belt: Meningitis Incidence and Carriage Prevalence in Children and Adults

**DOI:** 10.1371/annotation/38175137-0da2-4268-a959-c9ac06da9b3e

**Published:** 2013-10-10

**Authors:** Judith E. Mueller, Seydou Yaro, Macaire S. Ouédraogo, Natalia Levina, Berthe-Marie Njanpop-Lafourcade, Haoua Tall, Régina S. Idohou, Oumarou Sanou, Sita S. Kroman, Aly Drabo, Boubacar Nacro, Athanase Millogo, Mark van der Linden, Bradford D. Gessner

Labels along the x-axis were not printed completely. The corrected Figure 2C can be found here: 

**Figure:**