# Supplementary material for: Pneumococci in the African Meningitis Belt: Meningitis Incidence and Carriage Prevalence in Children and Adults
Source: PLoS One. 2012 Dec 20;7(12):e52464. doi: 10.1371/journal.pone.0052464 (PMC3527509; doi:10.1371/journal.pone.0052464)
Supplement: Table S1 — Algorithm of sequential multiplex polymerase chain reaction testing (PCR) for Streptococcus pneumoniae serotype determination for meningitis belt countries, with and internal positive control using cps A in each reaction. (DOCX) [file pone.0052464.s001.docx]

**Table S1**. Algorithm of sequential multiplex polymerase chain reaction testing (PCR) for *Streptococcus pneumoniae* serotype determination for meningitis belt countries, with and internal positive control using *cps*A in each reaction.

| Steps of multiplex PCR reactions | Serotypes included in each reaction | Size of PCR products  (pb) |
| --- | --- | --- |
|  | *cps*A | 160 |
| PCR 1 | 14  1  12A/12B/12F  25A/25F/38 | 189  280  376  574 |
| PCR 2 | 6A/6B  23F  4  18A/18B/18C/18F | 250  384  430  573 |
| PCR 3 | 19F  5  19A  35B  7F | 304  362  478  677  826 |
| PCR 4 | 7B/7C/40  3  35F/47F  10A  9A/9V | 260  371  517  628  753 |
| PCR 5 | 8  34  11A/11D  31 | 294  408  463  701 |
| PCR 6 | 33F  15B, 15C *  22F  16F | 338  496  643  988 |
| PCR 7 | 15A  20  17F | 436  514  693 |

x/y indicates primers that cross-react between several serotypes

* interconverting serotypes
