# Supplementary material for: Pneumococci in the African Meningitis Belt: Meningitis Incidence and Carriage Prevalence in Children and Adults
Source: PLoS One. 2012 Dec 20;7(12):e52464. doi: 10.1371/journal.pone.0052464 (PMC3527509; doi:10.1371/journal.pone.0052464)
Supplement: Table S2 — Characteristics of carriage study participants aged 1 month to 39 years (N = 519), by nasopharyngeal pneumococcal carriage status. Bobo-Dioulasso, February 2008. (DOCX) [file pone.0052464.s002.docx]

**Table S2**. Characteristics of carriage study participants aged 1 month to 39 years (N=519), by nasopharyngeal pneumococcal carriage status. Bobo-Dioulasso, February 2008.

| Category | Non-carrier (N=353*)  Number (%) in category | Carrier (N=166*)  Number (%) in category | Age-adjusted  *P*-value |
| --- | --- | --- | --- |
| Female sex | 202 (43%) | 90 (46%) | 0.99 |
| Malnutrition among children <9 years | 15/59 (25%) | 18/92 (20%) | 0.40 |
| Smoking among persons >14 years of age | 21/219 (10%) | 1/42 (2%) | 0.18 |
| Reported previous meningitis | 2 (1%) | 0 | N/A |
| Ever attended school among persons >9 years of age | 200/264 (76%) | 56/62 (90%) | 0.92 |
| Basic literacy | 157/264 (60%) | 35/62 (57%) | 0.68 |
| Television in the compound | 239 (69%) | 108 (65%) | 0.21 |
| Kitchen outside the house | 300 (85%) | 143 (87%) | 0.68 |
| ≥7 persons living in compound | 323 (92%) | 162 (98%) | 0.16 |
| ≥3 persons sharing sleeping room | 156 (44%) | 97 (58%) | 0.15 |
| Coughing observed by ENT nurse | 12 (3%) | 17 (10%) | 0.092 |
| Tonsillitis/pharyngitis observed by ENT nurse | 7 (2%) | 1 (1%) | 0.24 |
| Rhinorrhea observed by ENT nurse | 41 (12%) | 39 (23%) | 0.13 |
| Age: mean (standard deviation) | 18.2 (10.6) years | 9.1 (10.6) years | <0.001 (t-test) |

N (%)

* minor variations may occur due to missing values

*P*-values obtained from linear or logistic regression models, accounting for design effect and adjusting for age (<1y, 1-4y, 5-14yn 15-24y, 25-39y)
